# Liposarcoma of the tongue: case report and review of the literature

**DOI:** 10.1186/1746-160X-2-21

**Published:** 2006-07-26

**Authors:** Marika R Dubin, Edward W Chang

**Affiliations:** 1 Department of Otolaryngology – Head and Neck Surgery, Columbia University – New York Presbyterian Hospital, 180 Fort Washington Ave., HP 818, New York 10032, USA

## Abstract

**Background:**

Liposarcoma most commonly arises in the retroperitoneum and lower extremities. Liposarcoma of the head and neck is rare, with only 12 previously reported cases of liposarcoma in the tongue.

**Case presentation:**

We present a case of well-differentiated liposarcoma of the tongue occuring in a 39 year old man, treated with surgical excision. At 14 years of follow-up, the patient remains free of disease.

**Conclusion:**

Liposarcoma of the head and neck is rare, and may easily be misdiagnosed clinically. The diagnosis is made histologically. Clinical behavior is related to histopathologic subtype. Wide surgical excision is the treatment of choice, with limited data to support the use of radiation or chemotherapy. Our case represents the longest follow-up period for a tongue liposarcoma, with 14 years disease-free following surgical extirpation.

## Background

Liposarcoma is a malignant mesenchymal neoplasm that arises from adipose tissue, most commonly in the retroperitoneum and lower extremities. Liposarcoma of the head and neck is rare, representing 5.6% to 9% of cases in large series [1-3]. Common sites of occurrence in the head and neck region include the larynx, hypopharynx, oral cavity, orbit, scalp and soft tissues of the neck. In a recent review, Nikitakis et al [4] identified 44 cases of oral liposarcoma published in the English language literature between 1944 and 2001. Liposarcoma of the oral cavity demonstrates a predilection for the cheek [4-6], with other sites including the floor of the mouth, palate, gingiva, mandible, and tongue. To our knowledge, there have been only 12 previously reported cases of liposarcoma of the tongue in the English language literature (Table 1). We present a case of well-differentiated liposarcoma of the tongue and review the current literature.

**Table 1:** Cases of liposarcoma of the tongue published in the English language literature

**Authors**	**Age**	**Sex**	**Size**	**Histopathology**	**Follow-up**
Larson et al (1976) (11)	42	F	1.5 × 1.5 cm	WD	No follow-up available
Wescott and Correll(1984) (12)	61	M	3.5 × 3 × 2 cm	Myxoid, WD	No follow-up available
Guest (1992) (13)	71	M	1 cm	Myxoid, WD	2 y, NED
Minic (1995) (6)	68	F	2.5 × 1.5 × 1 cm	Myxoid, WD	3 y, NED
Saddik et al (1996) (14)	76	M	2.5 cm	WD	No follow-up available
Nelson et al (1998) (15)	37	M	3 × 3 × 3 cm	WD	1.5 y, NED
Gagari et al (2000) (9)	73	M	2 × 1 × 1 cm	WD lipoma-like	No follow-up available
Orita et al (2000) (16)	70	M	1 × 3.5 cm	WD lipoma-like	8 mo, NED
Moore et al (2001) (17)	43	M	8 mm	WD/Atypical lipoma	10 mo, NED
Nunes et al (2001) (18)	65	M	1 cm	WD	No follow-up available
Bengezi et al (2002) (19)	67	M	1.5 × 2.5 cm	Myxoid	2 y, NED
Capodiferro et al (2004) (20)	58	F	2.5 × 1.5 cm	WD	2 y, NED
Present Case	39	M	1.6 × 1.5 × 1.3 cm	WD	14 y, NED

WD = well-differentiated, NED = no evidence of disease

## Case presentation

**Figure 1:**
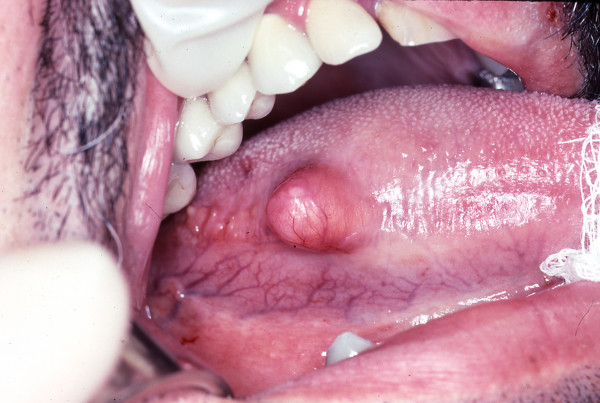
Gross appearance of the lesion on the right lateral aspect of the tongue.

**Figure 2:**
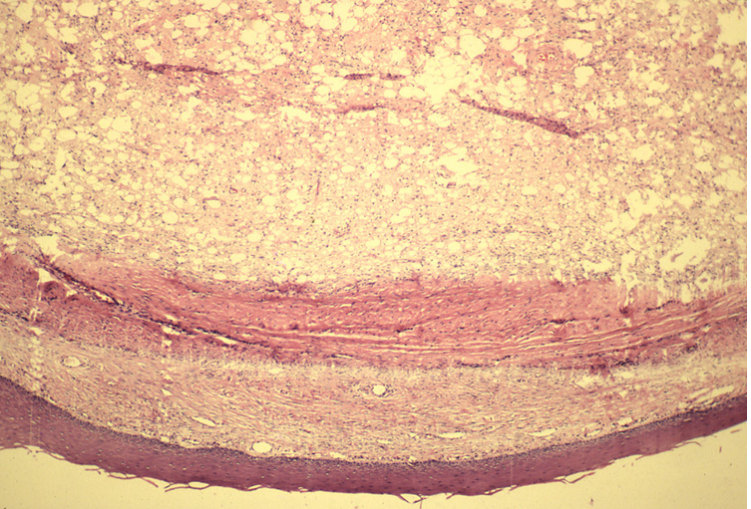
Low power view of well-differentiated liposarcoma demonstrating its well-circumbscribed nature (hematoxylin-eosin, ×2.5).

**Figure 3:**
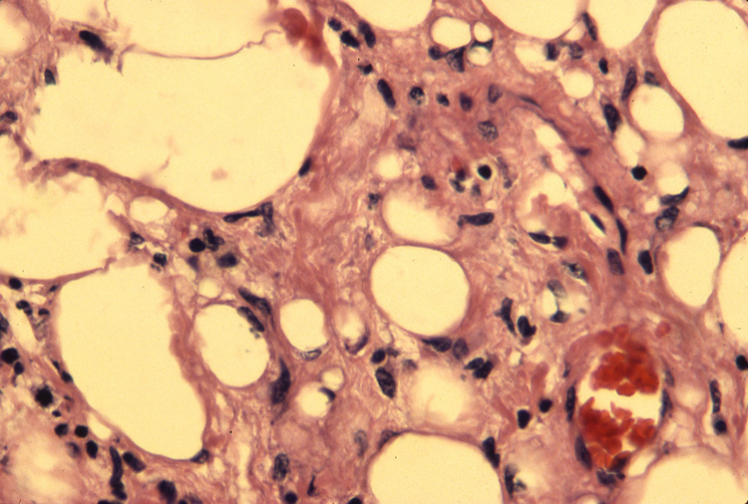
High power view demonstrating multiple lipoblasts in fibrous stroma. Note pleomorphic, hyperchromatic nuclei (hematoxyin-eosin, × 40).

A 39 year old man presented in late 1990 to an outside institution with a right lateral tongue mass. His past medical history was significant only for alcohol abuse and a history of syphilis. His physical examination revealed a 1 × 1 cm mass on the right lateral aspect of the tongue (Figure 1). The mass was felt to be a traumatic lesion secondary to abrasion against a fractured tooth. The fractured tooth was extracted. Six months later, the mass continued to enlarge in size and the patient presented to our institution. Preliminary diagnosis was fibroma and an excisional biopsy was performed. The pathologic specimen measured 1.6 × 1.5 × 1.3 cm in size. Microscopic examination revealed a well-circumscribed tumor composed of varying sized fat cells, fibrous tissue and numerous blood vessels. Numerous lipoblasts were present, and nuclei throughout the tumor were pleomorphic and hyperchromatic. Mitotic figures were present but rare (Figures 2 and 3). A diagnosis of well-differentiated liposarcoma was made. The case was presented to Tumor Board at Columbia Presbyterian Medical Center and the recommendation was for wide local excision of the previously biopsied area. The patient was taken back to the operating room and 1 cm margins of the previously biposied area were obtained with a KTP laser. Histologic inspection revealed no evidence of residual tumor. Fourteen years later, the patient remains free of disease. This is the longest follow-up of a tongue liposarcoma in the current literature.

## Conclusion

Liposarcoma demonstrates a peak occurrence between the 4^th ^and 6^th ^decade, with a slight male preponderance [2,3,7,8]. It typically presents as a painless, slowly enlarging mass, only becoming symptomatic when impinging upon surrounding structures [6,9,10]. In the majority of previously described cases of liposarcoma of the tongue, the only presenting feature was a painless mass [6,9,11-15,18-20]; one patient complained of local irritation against the teeth [17] and another complained of saliva dribbling from the mouth [16].

Liposarcoma can easily be misdiagnosed clinically. Its relatively indolent course often results in a misdiagnosis of cyst or benign soft tissue neoplasm; it is frequently mistaken for lipoma [12]. Liposarcoma has been described as more firm, less easily compressed and more fixed to adjacent tissue than lipoma, and on gross inspection, as less yellow and less lobulated [12,21]. Nonetheless, many authors report difficulty in distinguishing these entities [21,22] and therefore histopathology is required for an appropriate diagnosis [12,19]. The histologic characteristics that distinguish liposarcoma from intramuscular lipoma include the presence of lipoblasts, cellular pleomorphism, vascular proliferation and mitotic activity [30].

Liposarcoma is characterized by a variety of histologic variants that have been the subject of an evolving process of classification. Currently, the World Health Organization distinguishes the four variants proposed by Enzinger and Weiss based on developmental stage of the lipoblasts and overall degree of cellularity and pleomorphism [1]. These four entities are described as well-differentiated, myxoid, round-cell and pleomorphic. The WHO also recognizes a fifth variant, dedifferentiated, to describe changes occurring within well-differentiated liposarcoma that correspond with more aggressive clinical behavior and poor outcome [17,24]. The well-differentiated type has been further subclassified into lipoma-like, inflammatory, and sclerosing types [9].

Enzinger and Winslow demonstrated that histologic type correlates with clinical behavior; similar findings have been made in subsequent reviews [23,25,26]. Patients with well-differentiated and myxoid type tumors have higher 5-year survival rates and lower recurrence rates than patients with pleomorphic and round-cell types. The incidence of metastasis is also correlated with histologic type. Round-cell and pleomorphic types have higher rates of metastasis than well-differentiated and myxoid types, which almost never metastasize [1,3,7,23,26].

Wide surgical excision is the treatment of choice for liposarcoma. Recurrence rate increases from 17% to 80% with incomplete excision [26], as may occur when tumors are mistakenly believed to be benign lipomas [5]. Although grossly these tumors appear to be encapsulated, they extend by infiltration; the likelihood of nearby satellite nodules necessitates wide excision [11,31]. Lymph node dissection is not indicated unless there is concrete evidence of metastasis, since the likelihood of nodal metastases in this disease is so rare [24].

Nonsurgical treatment modalities are of limited use in liposarcoma. The use of radiation therapy remains controversial. Pack and Pierson [8] reported an increase in 5-year survival from 50% to 87% with combined surgery and radiation therapy compared to surgery alone. Evans' [32] review of 55 cases demonstrated a decrease in local recurrence for patients with myxoid liposarcoma treated with surgery followed by radiation compared to surgery alone; however, a significant difference in survival between the two groups was not shown. McCulloch et al [26] reported 11 cases where radiation therapy was used, 9 of them in conjunction with surgery. Only 4 patients were disease free at the end of follow-up. Just as the benefit of radiation therapy remains to be proven, there has been little data with regard to the usefulness of chemotherapy in treatment of liposarcoma.

Prognosis of liposarcoma is influenced by three factors: histologic variant, adequacy of surgical excision, and location of the tumor [9]. Golledge et al [27] found a relatively favorable prognosis for liposarcoma of the scalp, face and larynx as compared with the oral cavity, pharynx and neck, and attributed this difference to earlier recognition of tumor. Several authors have noted deep-seated liposarcoma of the head and neck to be associated with higher rates of recurrence, likely owing to incomplete excision secondary to cosmetic and physiologic considerations [9,10,14]. The differential clinical behavior of tumors by site has lead some authors to propose that well-differentiated tumors in superficial locations be classified as "atypical lipomas"; these tumors, while histologically similar to the well-differentiated type, are notable for their comparative ease of resection and decreased morbidity [22,29].

The role of tumor size in prognosis is unclear. Golledge et al [27] noted in their study of 76 patients that tumor size did not affect prognosis, however some authors have identified small size with better survival rate [3] and less risk of recurrence [28]. Several authors have commented that the prognosis of liposarcoma of the oral cavity is generally favorable because of the predominance of myxoid and well-differentiated types and the small size of these neoplasms. [6,16].

In summary, liposarcoma of the head and neck is rare, with only a handful of cases reported in the tongue. Liposarcoma of the tongue typically presents as a slow-growing painless mass, and may easily be mistaken for benign lipoma. Diagnosis is made histologically. The histopathologic variant influences clinical behavior and prognosis, with well-differentiated and myxoid tumors following a more benign course. The treatment of choice is wide surgical excision. The benefit of radiation and chemotherapy remains unproven. We present a case of a tongue liposarcoma with only surgical extirpation, and with a 14 year follow-up free of disease.

## Abbreviations

N/A

## Competing interests

The author(s) declare that they have no competing interests.

## Authors' contributions

EWC treated the patient and provided follow-up. MRD researched the current literature. MRD and EWC co-wrote the contents of this manuscript.
